# Supplementary material for: S100A4/FSP1: A Prognostic Marker and a Promising Target for Antitumor Therapy
Source: Int J Mol Sci. 2025 Sep 25;26(19):9370. doi: 10.3390/ijms26199370 (PMC12524625; doi:10.3390/ijms26199370)
Supplement: Supplementary file 1 [file ijms-26-09370-s001.zip › ijms-3864786-supplementary.pdf]

SUPPLEMENTARY MATERIALS

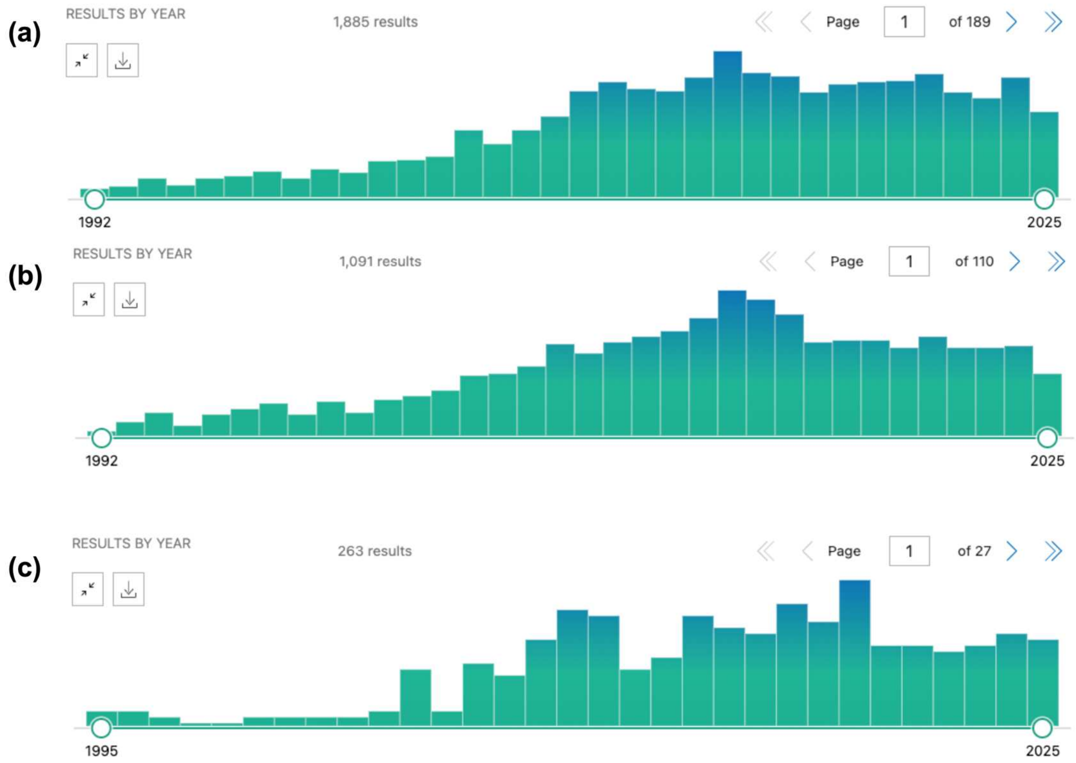

Figure S1. Number of publications cited in PubMed on request (a) «S100A4» (1885 results); (b) S100A4 cancer (1091 results); (c) S100A4 fibrosis (263).

Table S1. S100 calcium-binding protein A4: functional and physical associations, generated by STRING.

Gene	Function	STRING score
**ANXA2**	Annexin A2; Calcium-regulated membrane-binding protein whose affinity for calcium is greatly enhanced by anionic phospholipids. It binds two calcium ions with high affinity. May be involved in heat-stress response. Inhibits PCSK9-enhanced LDLR degradation, probably reduces PCSK9 protein levels via a translational mechanism but also competes with LDLR for binding with PCSK9 ; Belongs to the annexin family.	0.999
**MYH9**	Myosin-9; Cellular myosin that appears to play a role in cytokinesis, cell shape, and specialized functions such as secretion and capping. During cell spreading, plays an important role in cytoskeleton reorganization, focal contacts formation (in the margins but not the central part of spreading cells), and lamellipodial retraction; this function is mechanically antagonized by MYH10.	0.997
**TP53**	Cellular tumor antigen p53; Acts as a tumor suppressor in many tumor types; induces growth arrest or apoptosis depending on the physiological circumstances and cell type. Involved in cell cycle regulation as a trans-activator that acts to negatively regulate cell division by controlling a set of genes required for this process. One of the activated genes is an inhibitor of cyclin-dependent kinases. Apoptosis induction seems to be mediated either by stimulation of BAX and FAS antigen expression, or by repression of Bcl-2 expression	0.996
**AGER**	Advanced glycosylation end product-specific receptor; Mediates interactions of advanced glycosylation end products (AGE). These are nonenzymatically glycosylated proteins which accumulate in vascular tissue in aging and at an accelerated rate in diabetes. Acts as a mediator of both acute and chronic vascular inflammation in conditions such as atherosclerosis and in particular as a complication of diabetes. AGE/RAGE signaling plays an important role in regulating the production/expression of TNF-alpha, oxidative stress, and endothelial dysfunction in type 2 diabetes.	0.988
**FGF2**	Fibroblast growth factor 2; Acts as a ligand for FGFR1, FGFR2, FGFR3 and FGFR4. Also acts as an integrin ligand which is required for FGF2 signaling. Binds to integrin ITGAV:ITGB3. Plays an important role in the regulation of cell survival, cell division, cell differentiation and cell migration. Functions as a potent mitogen in vitro. Can induce angiogenesis.	0.869
**EGFR**	Epidermal growth factor receptor; Receptor tyrosine kinase binding ligands of the EGF family and activating several signaling cascades to convert extracellular cues into appropriate cellular responses. Known ligands include EGF, TGFA/TGF-alpha, AREG, epigen/EPGN, BTC/betacellulin, epiregulin/EREG and HBEGF/heparin- binding EGF. Ligand binding triggers receptor homo- and/or heterodimerization and autophosphorylation on key cytoplasmic residues. The phosphorylated receptor recruits adapter proteins like GRB2 which in turn activates complex downstream signaling cascades	0.858
**AREG**	Amphiregulin; Ligand of the EGF receptor/EGFR. Autocrine growth factor as well as a mitogen for a broad range of target cells including astrocytes, Schwann cells and fibroblasts; Belongs to the amphiregulin family.	0.822
**S100A1**	Protein S100-A1; Probably acts as a Ca(2+) signal transducer. In response to an increase in intracellular Ca(2+) levels, binds calcium which triggers a conformational change. This conformational change allows interaction of S1001A with specific target proteins, such as TPR-containing proteins, and the modulation of their activity. Belongs to the S-100 family.	0.807
**S100B**	Protein S100-B; Weakly binds calcium but binds zinc very tightly-distinct binding sites with different affinities exist for both ions on each monomer. Physiological concentrations of potassium ion antagonize the binding of both divalent cations, especially affecting high-affinity calcium-binding sites. Binds to and initiates the activation of STK38 by releasing autoinhibitory intramolecular interactions within the kinase. Interaction with AGER after myocardial infarction may play a role in myocyte apoptosis by activating ERK1/2 and p53/TP53 signaling.	0.757
**RHOA**	Transforming protein RhoA; Small GTPase which cycles between an active GTP-bound and an inactive GDP-bound state. Mainly associated with cytoskeleton organization, in active state binds to a variety of effector proteins to regulate cellular responses such cytoskeletal dynamics, cell migration and cell cycle. Regulates a signal transduction pathway linking plasma membrane receptors to the assembly of focal adhesions and actin stress fibers. Involved in a microtubule-dependent signal that is required for the myosin contractile ring formation during cell cycle cytokinesis.	0.732

**MDM2**	E3 ubiquitin-protein ligase Mdm2; E3 ubiquitin-protein ligase that mediates ubiquitination of p53/TP53, leading to its degradation by the proteasome. Inhibits p53/TP53- and p73/TP73-mediated cell cycle arrest and apoptosis by binding its transcriptional activation domain. Also acts as a ubiquitin ligase E3 toward itself and ARRB1. Permits the nuclear export of p53/TP53. Promotes proteasome-dependent ubiquitin-independent degradation of retinoblastoma RB1 protein. Inhibits DAXX-mediated apoptosis by inducing its ubiquitination and degradation.	0.706
**CDH1**	Cadherin-1; Cadherins are calcium-dependent cell adhesion proteins. They preferentially interact with themselves in a homophilic manner in connecting cells; cadherins may thus contribute to the sorting of heterogeneous cell types. CDH1 is involved in mechanisms regulating cell-cell adhesions, mobility and proliferation of epithelial cells. Has a potent invasive suppressor role. It is a ligand for integrin alpha-E/beta-7. (Microbial infection) Serves as a receptor for Listeria monocytogenes; internalin A (InlA) binds to this protein and promotes uptake of the bacteria.	0.666
**TGFB1**	Transforming growth factor beta-1 proprotein; Transforming growth factor beta-1 proprotein: Precursor of the Latency-associated peptide (LAP) and Transforming growth factor beta-1 (TGF-beta-1) chains, which constitute the regulatory and active subunit of TGF-beta-1, respectively. Transforming growth factor beta-1: Multifunctional protein that regulates the growth and differentiation of various cell types and is involved in various processes, such as normal development, immune function, microglia function and responses to neurodegeneration (By similarity).	0.660
**PPFIBP1**	Liprin-beta-1; May regulate the disassembly of focal adhesions. Did not bind receptor-like tyrosine phosphatases type 2A. Belongs to the liprin family. Liprin-beta subfamily.	0.651
**TLR4**	Toll-like receptor 4; Cooperates with LY96 and CD14 to mediate the innate immune response to bacterial lipopolysaccharide (LPS). Acts via MYD88, TIRAP and TRAF6, leading to NF-kappa-B activation, cytokine secretion and the inflammatory response. Also involved in LPS-independent inflammatory responses triggered by free fatty acids, such as palmitate, and Ni(2+). Responses triggered by Ni(2+) require non- conserved histidines and are, therefore, species-specific. Both M.tuberculosis HSP70 (dnaK) and HSP65 (groEL-2) act via this protein to stimulate NF-kappa-B expression.	0.643
**TWIST1**	Twist-related protein 1; Acts as a transcriptional regulator. Inhibits myogenesis by sequestrating E proteins, inhibiting trans-activation by MEF2, and inhibiting DNA-binding by MYOD1 through physical interaction. This interaction probably involves the basic domains of both proteins. Also represses expression of proinflammatory cytokines such as TNFA and IL1B. Regulates cranial suture patterning and fusion. Activates transcription as a heterodimer with E proteins. Regulates gene expression differentially, depending on dimer composition.	0.637
**TNF**	Tumor necrosis factor, membrane form; Cytokine that binds to TNFRSF1A/TNFR1 and TNFRSF1B/TNFBR. It is mainly secreted by macrophages and can induce cell death of certain tumor cell lines. It is potent pyrogen causing fever by direct action or by stimulation of interleukin-1 secretion and is implicated in the induction of cachexia, Under certain conditions it can stimulate cell proliferation and induce cell differentiation. Impairs regulatory T- cells (Treg) function in individuals with rheumatoid arthritis via FOXP3 dephosphorylation.	0.617
**MMP9**	67 kDa matrix metalloproteinase-9; May play an essential role in local proteolysis of the extracellular matrix and in leukocyte migration. Could play a role in bone osteoclastic resorption. Cleaves KiSS1 at a Gly-|-Leu bond. Cleaves type IV and type V collagen into large C-terminal three quarter fragments and shorter N-terminal one quarter fragments. Degrades fibronectin but not laminin or Pz-peptide. Belongs to the peptidase M10A family.	0.606
**CTNNB1**	Catenin beta-1; Key downstream component of the canonical Wnt signaling pathway. In the absence of Wnt, forms a complex with AXIN1, AXIN2, APC, CSNK1A1 and GSK3B that promotes phosphorylation on N-terminal Ser and Thr residues and ubiquitination of CTNNB1 via BTRC and its subsequent degradation by the proteasome. In the presence of Wnt ligand, CTNNB1 is not ubiquitinated and accumulates in the nucleus, where it acts as a coactivator for transcription factors of the TCF/LEF family, leading to activate Wnt responsive genes.	0.589
**EZR**	Ezrin; Probably involved in connections of major cytoskeletal structures to the plasma membrane. In epithelial cells, required for the formation of microvilli and membrane ruffles on the apical pole. Along with PLEKHG6, required for normal macropinocytosis.	0.585
**CD44**	CD44 antigen; Cell-surface receptor that plays a role in cell-cell interactions, cell adhesion and migration, helping them to sense and respond to changes in the tissue microenvironment. Participates thereby in a wide variety of cellular functions including the activation, recirculation and homing of T-lymphocytes, hematopoiesis, inflammation and response to bacterial infection. Engages, through its ectodomain, extracellular matrix components such as hyaluronan/HA, collagen, growth factors, cytokines or proteases and serves as a platform for signal transduction.	0.569

**SNAI2**	Zinc finger protein SNAI2; Transcriptional repressor that modulates both activator- dependent and basal transcription. Involved in the generation and migration of neural crest cells. Plays a role in mediating RAF1-induced transcriptional repression of the TJ protein, occludin (OCLN) and subsequent oncogenic transformation of epithelial cells (By similarity). Represses BRCA2 expression by binding to its E2-box- containing silencer and recruiting CTBP1 and HDAC1 in breast cells. In epidermal keratinocytes, binds to the E-box in ITGA3 promoter and represses its transcription.	0.557
**COL1A1**	Collagen alpha-1(I) chain; Type I collagen is a member of group I collagen (fibrillar forming collagen).	0.553
**ERBB3**	Receptor tyrosine-protein kinase erbB-3; Tyrosine-protein kinase that plays an essential role as cell surface receptor for neuregulins. Binds to neuregulin-1 (NRG1) and is activated by it; ligand-binding increases phosphorylation on tyrosine residues and promotes its association with the p85 subunit of phosphatidylinositol 3-kinase. May also be activated by CSPG5. Involved in the regulation of myeloid cell differentiation.	0.522
**AKT1**	RAC-alpha serine/threonine-protein kinase; AKT1 is one of 3 closely related serine/threonine-protein kinases (AKT1, AKT2 and AKT3) called the AKT kinase, and which regulate many processes including metabolism, proliferation, cell survival, growth and angiogenesis. This is mediated through serine and/or threonine phosphorylation of a range of downstream substrates.	0.517
**ZEB1**	Zinc finger E-box-binding homeobox 1; Acts as a transcriptional repressor. Inhibits interleukin-2 (IL-2) gene expression. Enhances or represses the promoter activity of the ATP1A1 gene depending on the quantity of cDNA and on the cell type. Represses E-cadherin promoter and induces an epithelial-mesenchymal transition (EMT) by recruiting SMARCA4/BRG1. Represses BCL6 transcription in the presence of the corepressor CTBP1. Positively regulates neuronal differentiation. Represses RCOR1 transcription activation during neurogenesis.	0.514
**IL1B**	Interleukin-1 beta; Potent proinflammatory cytokine. Initially discovered as the major endogenous pyrogen, induces prostaglandin synthesis, neutrophil influx and activation, T-cell activation and cytokine production, B- cell activation and antibody production, and fibroblast proliferation and collagen production. Promotes Th17 differentiation of T-cells. Synergizes with IL12/interleukin-12 to induce IFNG synthesis from T- helper 1 (Th1) cells.	0.506
**MMP2**	72 kDa type IV collagenase; Ubiquitinous metalloproteinase that is involved in diverse functions such as remodeling of the vasculature, angiogenesis, tissue repair, tumor invasion, inflammation, and atherosclerotic plaque rupture. As well as degrading extracellular matrix proteins, can also act on several nonmatrix proteins such as big endothelial 1 and beta- type CGRP promoting vasoconstriction. Also cleaves KISS at a Gly-|-Leu bond. Appears to have a role in myocardial cell death pathways. Contributes to myocardial oxidative stress by regulating the activity of GSK3beta.	0.498
**ERBB4**	Receptor tyrosine-protein kinase erbB-4; Tyrosine-protein kinase that plays an essential role as cell surface receptor for neuregulins and EGF family members and regulates development of the heart, the central nervous system and the mammary gland, gene transcription, cell proliferation, differentiation, migration and apoptosis. Required for normal cardiac muscle differentiation during embryonic development, and for postnatal cardiomyocyte proliferation. Required for normal development of the embryonic central nervous system, especially for normal neural crest cell migration.	0.484
**SPP1**	Osteopontin; Binds tightly to hydroxyapatite. Appears to form an integral part of the mineralized matrix. Probably important to cell-matrix interaction.	0.479
**CCL2**	C-C motif chemokine 2; Acts as a ligand for C-C chemokine receptor CCR2. Signals through binding and activation of CCR2 and induces a strong chemotactic response and mobilization of intracellular calcium ions. Exhibits a chemotactic activity for monocytes and basophils but not neutrophils or eosinophils. May be involved in the recruitment of monocytes into the arterial wall during the disease process of atherosclerosis.	0.479
**NRG1**	Pro-neuregulin-1, membrane-bound isoform; Direct ligand for ERBB3 and ERBB4 tyrosine kinase receptors. Concomitantly recruits ERBB1 and ERBB2 coreceptors, resulting in ligand-stimulated tyrosine phosphorylation and activation of the ERBB receptors. The multiple isoforms perform diverse functions such as inducing growth and differentiation of epithelial, glial, neuronal, and skeletal muscle cells; inducing expression of acetylcholine receptor in synaptic vesicles during the formation of the neuromuscular junction; stimulating lobuloalveolar budding and milk production in the mammary gland.	0.474
**ZEB2**	Zinc finger E-box-binding homeobox 2; Transcriptional inhibitor that binds to DNA sequence 5'-CACCT-3' in different promoters. Represses transcription of E-cadherin.	0.451

**AP2M1**	AP-2 complex subunit mu; Component of the adaptor protein complex 2 (AP-2). Adaptor protein complexes function in protein transport via transport vesicles in different membrane traffic pathways. Adaptor protein complexes are vesicle coat components and appear to be involved in cargo selection and vesicle formation. AP-2 is involved in clathrin-dependent endocytosis in which cargo proteins are incorporated into vesicles surrounded by clathrin (clathrin-coated vesicles, CCVs) which are destined for fusion with the early endosome	0.443
**HIF1A**	Hypoxia-inducible factor 1-alpha; Functions as a master transcriptional regulator of the adaptive response to hypoxia. Under hypoxic conditions, activates the transcription of over 40 genes, including erythropoietin, glucose transporters, glycolytic enzymes, vascular endothelial growth factor, HILPDA, and other genes whose protein products increase oxygen delivery or facilitate metabolic adaptation to hypoxia. Plays an essential role in embryonic vascularization, tumor angiogenesis and pathophysiology of ischemic disease.	0.435
**NR2C2**	Nuclear receptor subfamily 2 group C member 2; Orphan nuclear receptor that can act as a repressor or activator of transcription. An important repressor of nuclear receptor signaling pathways such as retinoic acid receptor, retinoid X, vitamin D3 receptor, thyroid hormone receptor and estrogen receptor pathways. May regulate gene expression during the late phase of spermatogenesis. Together with NR2C1, forms the core of the DRED (direct repeat erythroid-definitive) complex that represses embryonic and fetal globin transcription including that of GATA1.	0.432
**STAT3**	Signal transducer and activator of transcription 3; Signal transducer and transcription activator that mediates cellular responses to interleukins, KITLG/SCF, LEP and other growth factors. Once activated, recruits coactivators, such as NCOA1 or MED1, to the promoter region of the target gene. May mediate cellular responses to activated FGFR1, FGFR2, FGFR3 and FGFR4. Binds to the interleukin-6 (IL-6)-responsive elements identified in the promoters of various acute-phase protein genes. Activated by IL31 through IL31RA. Acts as a regulator of inflammatory response.	0.431
**MAPK3**	Mitogen-activated protein kinase 3; Serine/threonine kinase which acts as an essential component of the MAP kinase signal transduction pathway. MAPK1/ERK2 and MAPK3/ERK1 are the 2 MAPKs which play an important role in the MAPK/ERK cascade. They participate also in a signaling cascade initiated by activated KIT and KITLG/SCF. Depending on the cellular context, the MAPK/ERK cascade mediates diverse biological functions such as cell growth, adhesion, survival and differentiation through the regulation of transcription, translation, cytoskeletal rearrangements.	0.423
**AP2A2**	AP-2 complex subunit alpha-2; Component of the adaptor protein complex 2 (AP-2). Adaptor protein complexes function in protein transport via transport vesicles in different membrane traffic pathways. Adaptor protein complexes are vesicle coat components and appear to be involved in cargo selection and vesicle formation. AP-2 is involved in clathrin-dependent endocytosis in which cargo proteins are incorporated into vesicles surrounded by clathrin (clathrin-coated vesicles, CCVs) which are destined for fusion with the early endosome.	0.412
**ERBB2**	Receptor tyrosine-protein kinase erbB-2; Protein tyrosine kinase that is part of several cell surface receptor complexes, but that apparently needs a coreceptor for ligand binding. Essential component of a neuregulin-receptor complex, although neuregulins do not interact with it alone. GP30 is a potential ligand for this receptor. Regulates outgrowth and stabilization of peripheral microtubules (MTs). Upon ERBB2 activation, the MEMO1-RHOA-DIAPH1 signaling pathway elicits the phosphorylation and thus the inhibition of GSK3B at cell membrane.	0.407

Table S2. Expression and prognostic properties of S100A4 in solid tumors

Tumor type	Expression Compared to normal tissue	Type of cells	Correlation of prognostic properties	Proposed mechanisms of action in tumor progression	References
**Breast cancer**	High level	Tumor cells and CAFs	Poor prognosis, decreased overall and disease-free survival	Stimulation of invasion, EMT, angiogenesis, metastasis in bone	42-44, 46-50
**Endometrial cancer**	High level	Tumor cells and CAFs	Postoperative recurrence and the occurrence of metastases after surgery	Metastasis development	54
**Ovarian cancer**	High level	Tumor cells and CAFs	Tumor progression and chemoresistance	Anti-apoptotic mechanisms, EMT, cancer stem cells	56-58
**Colorectal cancer**	High level	Tumor cells and CAFs	Molecular marker for early prognosis, and chemoresistance, negative prognostic marker	Stimulation of invasion, metastasis development, migration, proliferation of tumor cells	60-69
**Gastric cancer**	High level	Tumor cells and CAFs	Correlation with tumor grade, stage, metastasis, invasion, and relapse, poor prognosis	Change of tumor microenvironment through increased angiogenesis and fibroblast activation	70-75
**Liver cancer**	High level	Exosomes	Poor prognosis, prognostic marker for metastasis development	Increased cell migration, invasion, and metastasis by activating the EMT process	76-77
**Pancreatic cancer**	High level	Tumor cells	Radioresistance, poor prognosis	Increased invasion	78-80
**Renal cancer**	High level	Tumor cells	Poor prognosis		81-82
**Thyroid cancer**	High level	Tumor cells	Correlation with tumor invasion and metastasis	Increased growth and metastasis of tumor	83-85
**Eye cancer**	High level	Tumor cells	Decreased survival rate	Metastasis formation	87-92
**Head and neck cancer**	High level	Tumor cells, mesenchymal cells	High patient survival	Decreased progression	94
**Brain cancer**	High level	Tumor cells	Tumor progression	EMT, cancer stem cells	95-98
**Lung cancer**	High level	Tumor cells	Poor prognosis	Increased proliferation, migration, invasion and apoptosis	99-101

Table S3. S100A4 mediated signaling pathways involved in oncogenesis

Signaling pathway	Type of tumor	Mechanism of the influence on oncogenesis	References
**RAGE signaling**	Colorectal cancer	Hypoxia	63, 107-109, 111, 112
	Thyroid cancer	Metastasis	
	Melanoma	Metastasis	
	Breast cancer	Invasion, vascularization EMT, proliferation of tumor cells	
	Prostate	Metastasis	
	Bladder	Proliferation, metastasis of tumor cells	
	Lung	Proliferation, metastasis of tumor cells	
**Annexin A2**	Gastric cancer	Metastasis	118
**NMIIA**	Glioblastoma	Migration, hypoxia, angiogenesis, vascularization	97, 129, 130
	Endometrial cancer	Migration, stemness	

	Ovarian cancer	EMT, stemness	
**Wnt/β-catenin**	Colorectal cancer	Migration, invasion	39, 136
	Prostatic hyperplasia	Fibrosis, proliferation	
**Metalloproteases**	Uveal melanoma	Invasion, metastases	91, 138, 139, 163
	Breast cancer	Stemness	
**FGF2/FGFR1**	Breast cancer	Angiogenesis, migration of CAFs	141, 142
**p53**	Rectal carcinoma	Chemoresistance	147
**TGFβ1/ SMAD**	Endometrial cancer	Motility, invasion, fibrosis	24, 51
**MTA1-S100A4-NMIIA**	Prostate cancer	Angiogenesis	152, 154
**Sonic hedgehog-Gli1**	Pancreatic cancer	EMT	158
**E-cadherin (loss), β-catenin**	Vater cancer	EMT, invasion	160
**TGFβ1/(LASP1)/S100A4/Smad**	Colorectal cancer	EMT, invasion	161
**IKK/NF- κB/TNFalpha/IL2**	Bladder cancer	Stemness	159
**SNAIL2 and ZEB**	Glioma	Stemness	96

Table S4. The side effects of the drugs targeting the S100A4 protein.

Drug	List of side effects
**Trifluoperazine**	Drowsiness, dizziness, dry mouth, sleep disorders, fatigue, visual disturbances; extrapyramidal disorders, tardive dyskinesia; anorexia, cholestatic jaundice; thrombocytopenia, anemia, granulocytopenia, pancytopenia; tachycardia, moderate orthostatic hypotension, cardiac arrhythmias, ECG changes (QT prolongation, T wave smoothing); skin rash, urticaria, angioedema; galactorrhea, amenorrhea.
**Niclosamide**	Dyspeptic symptoms, allergic reactions, pruritus, exacerbation of neurodermatitis
**Sulindac**	NSAID gastropathy: nausea, abdominal pain, heartburn, diarrhea; bleeding: gastrointestinal, gingival, hemorrhoidal, uterine; bronchospasm, heart failure, edema, hypertension, headache, dizziness, photophobia, hepatic-renal failure, agranulocytosis, leukopenia, anemia, allergic reactions.
**Protac**	Experimental drug, no data on side effects.
**Amlexanox**	Mild painful tingling, burning, nausea, diarrhea.
**Pentamidine**	When used in inhalations, cough, shortness of breath, bronchospasm, rash, fever, anorexia, metallic taste in the mouth, fatigue, dizziness are possible; with parenteral administration of the drug, arterial hypotension, syncope, nausea, increased urea and creatinine in blood plasma, hyperkalemia, hematuria, proteinuria, hypoglycemia followed by hyperglycemia, acute pancreatitis, leukopenia, anemia, thrombocytopenia are possible.
